# High Prevalence of *Salmonella* spp. in Ready-to-Eat Artisanal Pork Sausages Sold at Food Outlets in Quindío, Colombia

**DOI:** 10.3390/pathogens14010031

**Published:** 2025-01-04

**Authors:** Elizabeth Jaramillo-Bedoya, Liliana Janeth Flórez-Elvira, Iván Darío Ocampo-Ibáñez

**Affiliations:** 1 Laboratorio de Salud Pública Departamental, Secretaría de Salud Departamental del Quindío, Gobernación del Quindío, Armenia 630008, Colombia; lizzyjaramillo88@gmail.com; 2 School of Basic Sciences, Faculty of Health, Universidad del Valle, Cali 760042, Colombia; liliana.florez@correounivalle.edu.co; 3 Research Group of Microbiology, Industry and Environment (GIMIA), Faculty of Basic Sciences, Universidad Santiago de Cali, Cali 760035, Colombia

**Keywords:** foodborne diseases, non-paratyphoid *Salmonella* spp., artisanal pork sausages

## Abstract

Non-typhoidal salmonellosis is a foodborne disease caused by *Salmonella* spp. Most outbreaks of this disease are commonly associated with consuming contaminated meat products, hence the importance of monitoring ready-to-eat artisanal pork sausages for the presence of these bacteria. A total of 494 samples of grilled and smoked barbecue artisanal pork sausages were collected at food outlets from 12 municipalities of the Department of Quindío, Colombia, between 2017 and 2022. *Salmonella* spp. was identified using VIDAS^®^ Easy SLM and confirmed through API^®^ 20 E. *Salmonella* spp. was detected in 260 samples (52.6%), and the highest rates of contamination were found in Armenia (65.7%), Salento (65.2%), Circasia (57.7%), and Calarcá (56.4%). The highest proportion of these samples positive for Salmonella spp. was ready-to-eat smoked barbecue artisanal sausages (68.8%) sold by street vendors (58.4%) from 12 municipalities of the Department of Quindío. A significant association was observed between the municipality and contamination of samples with *Salmonella* spp. However, no link was found between the sampling year and the presence of the bacteria. This is the first study aimed at monitoring the presence of *Salmonella* spp. in artisanal pork sausages sold in the municipalities of the Department of Quindío over a 6-year period, and findings revealed very high percentages of contamination. Although Colombian legislation establishes sanitary and safety requirements for meat production, the presence of *Salmonella* spp. in artisanal sausages remains a persistent public health threat in developing countries.

## 1. Introduction

Non-paratyphoid *Salmonella* spp. are Gram-negative bacteria that cause non-typhoidal salmonellosis, a foodborne disease that poses a worldwide public health problem [1]. According to the White–Kauffmann–Le Minor scheme, this group of *Salmonella* comprises >2600 serotypes, approximately 1600 of which belong to the species *Salmonella* enterica [2,3]. Many serotypes can cause digestive disorders in humans; however, *S. enterica* serovar Enteriditis and *S. enterica* serovar Typhimurium are the two most common serotypes causing human non-typhoidal salmonellosis and are thus of significant public health relevance [3,4].

Non-typhoidal salmonellosis usually presents as an acute diarrheal illness characterized by gastrointestinal symptoms such as diarrhea, fever, intestinal cramps, nausea, and vomiting lasting up to 7 days [5]. Although most cases of non-typhoidal salmonellosis are mild, the disease can occasionally be fatal due to serious complications such as bloodstream infections and focal infections. Children aged <5 years, adults >65 years, and immunocompromised individuals are at the highest risk [1,5]. The incidence of this disease is considered moderate, with an estimated 150 million cases and between 60,000 and 150,000 deaths worldwide every year [6]. It is a notifiable disease in Colombia, with an estimated 200,000–400,000 cases occurring annually [7]. In recent years, several outbreaks of non-typhoidal salmonellosis linked to the consumption of contaminated food have been reported in North and South America, Africa, Asia, and Europe [3,8,9,10,11]. The largest global outbreak of this disease to date was associated with a strain of *Salmonella typhimurium* linked to Belgian chocolate products [8]. A total of 151 genetically related cases were reported across 11 countries, potentially linked to the consumption of these chocolate products [8]. Numerous outbreaks of non-typhoidal salmonellosis associated with consuming contaminated food or water have been reported in Colombia in recent years, with a national average morbidity rate of 21.01 cases per 100,000 individuals [12].

Most outbreaks of non-typhoidal salmonellosis are usually associated with consuming animal-derived food products contaminated with *Salmonella* spp., primarily including eggs, meat, poultry, and dairy products [1,13]. Due to their ability to adapt to diverse environments and conditions, these bacteria can contaminate various types of food [14]. Although warm weather and non-refrigerated food provide ideal growth conditions [15], it has been observed that *Salmonella* spp. can adapt and grow under extreme conditions, with temperatures ranging from 2 °C to 54 °C and pH values between 3.8 and 9.5 [14,16]. Thus, they can multiply under conditions commonly used to preserve fresh or processed foods at room temperature and under refrigeration [14,16]. In particular, meat products that are consumed raw and produced in an artisanal manner have also been associated with non-typhoidal salmonellosis outbreaks, as they are often left to stand at room temperature, which facilitates the growth of *Salmonella* [15,17]. Furthermore, factors such as the product formulation, transportation without refrigeration from the store to the consumer’s home, and inadequate storage at home can also contribute to the growth of *Salmonella* spp. in these raw meat products [18]. However, *Salmonella* spp. has also been detected in ready-to-eat meat, pork, and poultry products, despite being grilled, cooked, fried, or barbecued [18,19,20,21]. This bacteria can be found in this kind of food due to several factors such as cross-contamination, improper cooking, poor hygiene practices, and contaminated ingredients [18,19]. In many cases, artisanal pork products such as sausages, ham, bacon, and meat pies are homemade or produced informally, with poor food-handling practices and without complying with established sanitary regulations [14,22]. In most developing countries, these products are sold by street vendors, but serious concerns exist about their handling and safety, and therefore persistent concerns about health risks, mainly due to unregulated practices [23].

Many outbreaks of non-typhoidal salmonellosis have been linked to consuming artisanal pork sausages around the world. The primary risk factors associated with cross-contamination with *Salmonella* in these meat products include manufacturing conditions, sanitary and handling practices, storage, and contamination of the raw meat used as raw material [16,17,22,24,25]. In Colombia, even though regulations establish sanitary and safety requirements for the preparation of meat products [26,27,28], fresh raw artisanal sausages (known as *chorizos artesanales*) have been identified as the foods with the highest rates of *Salmonella* contamination [12,29]. Fresh raw artisanal sausages are meat products made from a mixture of raw pork, pork or beef fat, and seasonings (salt, garlic, oregano, and pepper) that are stuffed into natural pork casings [30]. In the department of Quindío, most of these products are homemade, and therefore no microbiological control is carried out on the raw materials or the finished products [12,29,30]. In addition, inadequate storage and poor food-handling practices are among the main risk factors associated with the contamination of these products [12,29]. According to a study conducted in the Caribbean region of Colombia, the prevalence of *Salmonella* spp. in artisanal sausages was 13%, while in other foods, such as raw meat, eggs, and chicken, it did not exceed 7%, likely due to the storage of the products at room temperature [12,29]. However, the actual prevalence of *Salmonella* spp. in ready-to-eat grilled, smoked, or fried artisanal sausages produced in artisanal conditions and sold throughout Colombia, particularly in the Department of Quindío, where these meat products are produced and consumed on a large scale, remains unknown. In Quindío, the ready-to-eat artisanal sausages are most commonly served in food outlets, especially in restaurants, quick-service restaurants, by street vendors, hotels, and even small outlets that involve different styles of preparations. In this region, most artisanal sausages are sold by street vendors, located in open spaces or along the streets, where they are exposed to the environment and without regulation or monitoring by the government authorities. Therefore, this study aimed to determine the presence of *Salmonella* spp. in artisanal sausages sold in food outlets across the 12 municipalities of the Department of Quindío between 2017 and 2022. Knowing the prevalence of *Salmonella* spp. in ready-to-eat artisanal sausages will provide important insights for preventing and controlling contamination with foodborne pathogenic bacteria. As the first study on the presence of *Salmonella* spp. in ready-to-eat artisanal sausages marketed in the Department of Quindío, Colombia, it will provide key data to enhance the control and surveillance measures established by the Colombian legislation, which regulates the sanitary and safety requirements for the production of these foods, as well as generate an alarm regarding the public health risk of the consumption of these foods that are produced in an artisanal manner and sold in food outlets.

## 2. Materials and Methods

### 2.1. Sampling

This study was conducted between February 2017 and March 2022 in the Department of Quindío, located in Central–Western Colombia between 04°04′41″ N–04°43′18″ N and 75°23′41″ W–75°53′56″ O. Samples of artisanal “santarrosano” sausages were randomly collected from various ready-to-eat food outlets, including restaurants, quick-service restaurants, and street vendors, in the 12 municipalities of the Department of Quindío, namely Armenia, Buenavista, Calarcá, Circasia, Córdoba, Filandia, Génova, La Tebaida, Montenegro, Pijao, Quimbaya, and Salento. A cross-sectional design was used yearly, with a total non-probability convenience sampling of 494 ready-to-eat artisanal sausage samples (each weighing 100 and 150 g), including grilled, fried, or smoked barbecue sausages. Some differences in the number of samples for municipalities were found because most of the municipalities in the Department of Quindío are very small and located in rural areas. Consequently, many of them have only one food outlet for ready-to-eat artisanal sausages, making the availability and access to sample collection in these areas quite difficult. Despite all the artisanal sausages being made under artisanal conditions, the information about the origin of ingredients and production conditions was not available. The Departmental Public Health Laboratory of Quindío carried out sample collection as part of the laboratory surveillance procedures.

### 2.2. Isolation and Identification of Salmonella spp.

The presence of *Salmonella* spp. in the samples was determined according to the ISO standards [31] and the guidelines of the Food and Drug Administration [32]. The detection of *Salmonella* spp. was carried out using the commercial kit VIDAS^®^ Easy SLM (bioMérieux, Marcy l’Etoile, France) following the manufacturer’s instructions. Briefly, 25 g of artisanal sausage was homogenized and selectively enriched for *Salmonella* spp. in 225 mL buffered peptone water (bioMérieux) and incubated at 37 °C for 22 h. Next, 0.1 mL of this suspension was transferred to 10 mL of *Salmonella* Xpress 2 selective broth (bioMérieux) and incubated at 41.5 °C for 22 h. Subsequently, 500 μL was used for detection in the VIDAS^®^ system. From suspensions containing *Salmonella* spp., 10 μL were taken for colony isolation on ChromID™ *Salmonella* selective media (bioMérieux) and incubated at 37 °C for 24 h. Finally, the colonies obtained were confirmed by biochemical tests using the API^®^ 20E kit (bioMérieux) according to the manufacturer’s instructions. All assays were performed in triplicate, and at least two independent assays were performed for each sample of artisanal sausages.

### 2.3. Statistical Analysis

The frequency distribution of qualitative variables was determined through univariate and bivariate analyses using contingency tables. The distribution of *Salmonella* spp. relative to the municipality of origin of the artisanal sausage and the year was determined. Statistical significance was evaluated using Fisher’s exact test with a significance level of 0.05. All analyses were performed using the R-Project statistical package, version 4.3.1.

## 3. Results

**Figure 1:**
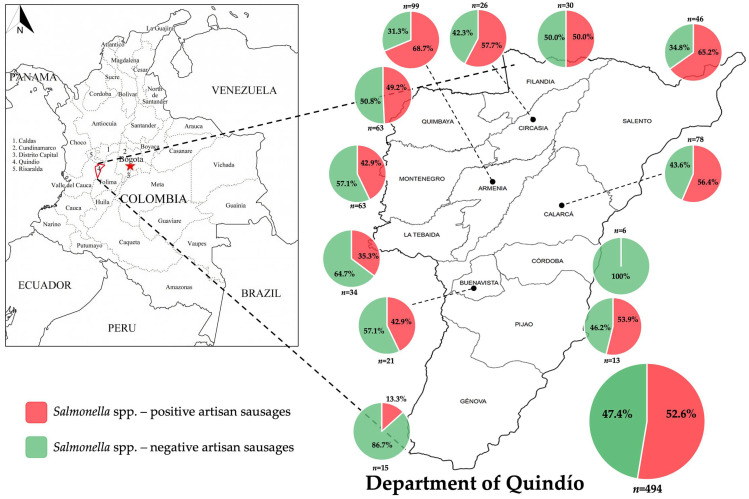
Cumulative prevalence of *Salmonella* spp. in the Department of Quindío over 6 years under study.

This study aimed to determine the presence of *Salmonella* spp. in ready-to-eat artisanal pork sausages sold across the Department of Quindío between 2017 and 2022. A total of 494 samples of ready-to-eat grilled, fried, or smoked barbecue artisanal sausages were collected during this 6-year period from the 12 municipalities evaluated (Figure 1). *Salmonella* spp. was detected in 260 samples (prevalence of 52.6%) from 11 municipalities out of the 12 districts under study (Figure 1). The prevalence of *Salmonella* spp. over 6 years evaluated here varied significantly among the municipalities of Quindío (Figure 1), ranging from 13.3% in Génova to 68.7% in Armenia (Figure 1). The highest prevalence values were observed in Salento (65.2%), Circasia (57.7%), and Calarcá (56.4%) (Figure 1). The municipalities with the lowest prevalence of *Salmonella* spp. were La Tebaida (35.3%) and Génova (13.3%). Meanwhile, none of the samples from Córdoba tested positive for *Salmonella* spp. (prevalence of 0%) (Figure 1). Comparative analyses showed significant differences in the distribution of samples positive and negative for *Salmonella* spp. relative to the municipality (Figure 1).

In the analysis of the annual prevalence of *Salmonella* spp. in ready-to-eat artisanal pork sausages in the Department of Quindío, Fisher’s test showed no significant differences in the distribution of samples positive and negative for *Salmonella* spp. in relation to the year (Table 1). The highest proportion of positive samples was found in 2017 (56.5%) and 2022 (55.1%) (Table 1), while the highest number of samples negative for *Salmonella* spp. was observed in 2021 (50.6%) and 2020 (49.4%) (Table 1).

Based on the analysis of the proportion of sausages contaminated with *Salmonella* spp. annually in each municipality over the 6-year period under study, the highest number of positive samples was found in Armenia (2017, 17 samples; 2021, 14 samples) and Calarcá (2021, 8 samples; 2022, 8 samples) (Table 2). Pijao, Génova, and Buenavista had the lowest number of contaminated samples every year, with a maximum of two positive samples in 2017, 2019, and 2022 (Table 2). Córdoba was the only municipality with no samples positive for *Salmonella* spp. over these 6 years (Table 2). Finally, regarding the annual prevalence of *Salmonella* spp. in each municipality, 11 of the municipalities of the Department of Quindío (all except Córdoba) had prevalence values above 25% in at least one of the years evaluated (Table 2). Armenia, Buenavista, Calarcá, Circasia, Filandia, and Salento presented extremely high prevalence of *Salmonella* spp. in artisanal sausages, some years even exceeding 60% (Table 2). The highest prevalence values were observed in Armenia in 2022 (76%) and 2021 (73%), and Salento in 2022 (71%) (Table 2).

The artisanal sausages marketed in three typical ready-to-eat food outlets analyzed in this study, restaurants, quick-service restaurants, and street vendors, presented different prevalence values for Salmonella spp. (Table 3). In this regard, the ready-to-eat artisanal sausages from street vendors were the most frequently contaminated products (67.6%) (Table 3). Among the positive samples, sausages from street vendors and quick-service restaurants showed the highest percentages of contamination, 58.4% and 25.8%, respectively (Table 3). Fisher’s test showed significant differences in the positive and negative samples’ behavior for Salmonella spp. in relation to the place of sale (Table 3).

Comparison analysis showed significant differences in the distribution of positive and negative samples for Salmonella spp. about the types of cooking of artisanal sausages (Table 4). Of the 260 samples positive for Salmonella spp., the highest proportion of contaminated samples was found for smoked sausages (68.8%) (Table 4), and among the samples negative for Salmonella spp., fried sausages accounted for the highest proportions of non-contaminated samples of 42.3% (Table 4).

## 4. Discussion

This is the first study conducted to determine the annual prevalence of *Salmonella* spp. in ready-to-eat cooked artisanal pork sausages sold in food outlets in the municipalities of the Department of Quindío, Colombia. The overall prevalence observed for Quindío over the 6-year period under study was extremely high (52.6%) and significantly higher than the average prevalence reported for ready-to-eat pork sausages in regions of some countries in Asia (China, 0.56%) [33], Europe (0.07% in Italy, 11.1% in Spain, and 8.6% in the United Kingdom) [34,35], South America (Brazil, 24.4%) [24,36], and North America (United States, 0.74%) [18]. Nevertheless, our results are consistent with data previously reported in certain North American countries, including Mexico, where *Salmonella* spp. prevalence in pork sausages reached up to 41% [37]. Even though legislation establishes sanitary and safety requirements for producing meat products, including pork sausages [26,27,28], reports on the surveillance and prevalence of *Salmonella* spp. in this type of food in Colombia are scarce [38]. In this regard, only a few studies have reported the prevalence of these bacteria in artisanal sausages in other departments and regions of Colombia [29,38,39]. The overall prevalence observed in the present study was higher than the previously reported values for other departments, such as Atlántico, Bolívar, and Córdoba (ranging from 2% to 12.6%), as well as Bogotá D.C. (ranging from 8% to 28%) [29,39,40,41].

Compared to previous studies conducted in Colombia [29,39,40,41], the overall prevalence of *Salmonella* spp. observed is remarkably high, particularly in some municipalities such as Armenia, Salento, Circasia, and Calarcá, where values exceeded 56% (Figure 1). Notably, Armenia, Calarcá, and Circasia have the highest population densities in the department, which implies a higher risk of potential outbreaks associated with consuming ready-to-eat artisanal sausages in these municipalities. In contrast, the lowest prevalence was found in La Tebaida and Génova (below 35%) and Córdoba (0%) (Figure 1). Significant differences were observed in the prevalence of *Salmonella* spp. relative to the municipality, indicating a relationship between the presence of the bacteria in ready-to-eat artisanal pork sausages and the municipality of the Department of Quindío where they are sold (Figure 1).

Ready-to-eat artisanal meat products are among the major public health concerns due to their elevated risk of *Salmonella* spp. contamination [15,17]. Many factors could be hazards for artisanal meat products, including initial contamination of raw foods at preparation and subsequent contamination by vendors during handling [42]. The primary risk factors associated with cross-contamination with *Salmonella* spp. in these products during preparation include unhygienic conditions, improper food-handling practices by manufacturers, transportation without refrigeration, inadequate storage, and contamination of the raw meat used as raw material [15,16,17,22,24,25]. Despite the lack of information on the manufacturing conditions of the products analyzed in our study, it is known that many meat products manufactured and sold in the Department of Quindío are homemade or artisan-produced in an informal way at home in rural areas. Therefore, the contamination observed here could be attributed to poor handling of raw materials and non-compliance with established sanitary standards for production [14,22] or using contaminated raw materials [16]. The facilities used for manufacturing sausages sold in the Department of Quindío may not meet the sanitary conditions required by Colombian regulations, among others, having minimum biosecurity measures to avoid direct and indirect cross-contamination [26,27,28].

On the other hand, poor and improper sanitary and hygienic practices during food preparation and handling are key factors for the transmission of pathogens from food handlers to consumers [23,43]. In this respect, several studies have found that food handlers can significantly contribute to the transmission of foodborne bacterial pathogens by using improper food-handling practices [42,43,44]. In particular, *Salmonella* spp. are commonly detected in street-vended foods due to unsanitary and improper food-handling [23,42]. In our study, we found significant differences in the prevalence of *Salmonella* spp. regarding the food outlets analyzed. High levels of *Salmonella* spp. contamination were found in the ready-to-eat cooked artisanal pork sausages sold by the street vendors in the Department of Quindío between 2017 and 2022; however, restaurants and quick-service restaurants also had some samples positive for *Salmonella* spp. These results could be due to improper handling practices by food handlers or vendors in some food outlets evaluated in this study. Although this study did not analyze the manufacturing process and conditions, the microbiological quality of raw materials, the biosafety practices of handlers, or the handling stages of the artisanal pork sausages, inadequate hygiene conditions and improper handling practices were observed at the moment of sampling, such as not using essential personal protective equipment, displaying raw and cooked sausages in the same places, using the same equipment and utensils meant for raw and cooked sausages, and infrequent washing of utensils. In this respect, the food handler could directly cross-contaminate the artisanal sausages during preparation by allowing raw sausages to come in contact with ready-to-eat cooked sausages or allowing juices to flow from raw to cooked sausages [14], or handlers with poor personal hygiene standards could indirectly contaminate foods by touching cooked sausages without prior washing of hands [14,23,42]. This hypothesis is supported by reports indicating that the prevalence of *Salmonella* spp. among food handlers in Colombia is notably high [45,46,47]. For example, a prevalence of up to 25% among meat food handlers has been reported in cities such as Villavicencio, Bucaramanga, Barranquilla, Pasto, and Bogotá D.C. [45,46,47]. Our results about the prevalence of *Salmonella* spp. in cooked artisanal sausages sold by street vendors were surprisingly higher than those previously reported in other developing countries, such as Ghana, where *Salmonella* spp. prevalence in grilled sausages reached up to 18.75% [43,44].

Thermal treatment is one of the more effective food-processing techniques to eliminate foodborne pathogens from food products, including *Salmonella* spp. [19]. However, some strains of this bacteria are capable of growing at high temperatures and thus may survive the thermal processing of some foods; therefore, *Salmonella* spp. can be detected in some ready-to-eat foods [19,21,33,44]. In this respect, we found that the thermal treatment by frying, grilling, and smoking on the barbecue could have been effective in eliminating any *Salmonella* spp. from 47.4% of artisanal sausages, but this bacterium was detected in 52.6% of the ready-to-eat cooked artisanal sausages here analyzed. However, as mentioned before, we did not consider the manufacturing process and conditions to evaluate the microbiological quality of raw artisanal sausages and confirm if the thermal treatment was or was not effective in eliminating *Salmonella* spp. Despite this, we found significant differences in the distribution of positive and negative samples for *Salmonella* spp. concerning types of cooking of artisanal sausages, which could suggest that in the Department of Quindío, *Salmonella* spp. is more likely to be found among smoked sausages. Most of the smoked barbecue sausages analyzed in this study came from street vendors, which would support the hypothesis that the high presence of *Salmonella* spp. is mainly due to direct or indirect cross-contamination caused by improper handling practices by food handlers. In Colombia, street vendors are considered a public health problem, as they have become a significant risk factor for the health of all consumers due to the poor and limited hygiene and cleanliness conditions in most of the sales stands [48]. Our results show that the prevalence of *Salmonella* spp. in ready-to-eat smoked barbecue sausages sold by street vendors found in this study was higher than what was previously reported in other cities of Colombia, such as Bogotá, where *Salmonella* spp. prevalence in several types of ready-to-eat smoked barbecue food ranged from 11.5% to 30% [40,41,48,49].

Finally, when comparing the prevalence values between 2017 and 2022, no significant differences were observed in the distribution of samples positive and negative for *Salmonella* spp. in relation to the year. However, the highest proportions of contaminated sausages were found in 2017 and 2018. When analyzing the prevalence in each municipality, Armenia (in 2021 and 2022) and Salento (in 2022) showed the highest prevalence, while Córdoba had no positive samples for *Salmonella* spp. Throughout the 6 years assessed in this study. Our results on the prevalence from 2017 to 2022 suggest that the poor sanitary conditions in producing artisanal sausages have been a long-standing issue, which may have significantly contributed to the high proportions and prevalence of *Salmonella* spp. detected in the artisanal sausages sold in the Department of Quindío.

Estimates of the prevalence of *Salmonella* spp. in ready-to-eat cooked artisanal pork sausages sold in the Department of Quindío were included in this study, considering the municipality, the year of selling, food outlets, and types of cooking, but the sanitary and safety conditions for producing and handling these products, such as the quality of the raw materials during production, transport, storing, and manufacturing practices for sausages, were not considered, which is a limitation. Nevertheless, it is important to highlight that this is the first study conducted with this objective, and strikingly high prevalence values of *Salmonella* spp. were detected in this region of Colombia. Considering that current Colombian legislation establishes the sanitary and safety requirements for edible meat products for human consumption and given the serious consequences that infection with these bacteria can have on human health, this study aims to raise awareness of the need to enhance and implement new strategies for the surveillance and control of *Salmonella* spp. in producing and commercializing artisanal pork sausages.

## 5. Conclusions

Our estimates of *Salmonella* spp. prevalence from 2017 to 2022 confirm that ready-to-eat fried, grilled, and smoked barbecue artisanal pork sausages produced and sold in restaurants, quick-service restaurants, and street vendors in the municipalities of the Department of Quindío are frequently contaminated with these bacteria. The results suggest that the conditions for producing and commercializing these meat products in the department do not comply with the minimum sanitary and safety requirements established by Colombian legislation for edible meat products intended for human consumption. Since pork sausages sold nationwide are produced in artisanal settings, it is essential to replicate this type of study in other departments of Colombia. Our results highlight the urgent need to enhance and design new strategies for the surveillance and control of *Salmonella* spp. in food production and commercialization at both the departmental and national levels, considering the risk this pathogen poses to human health, particularly for high-risk groups such as children, adults, and immunocompromised individuals. The high prevalence of *Salmonella* spp. in artisanal sausages sold by street vendors in the Department of Quindío confirms this public health problem because they have become a significant risk factor for the health of all consumers due to the poor and limited hygiene and cleanliness conditions in most of the sales stands.

## Figures and Tables

**Table 1:** The proportion of ready-to-eat artisanal sausage samples positive and negative for *Salmonella* spp. over the different years under study.

Year ^1^	Negative Samples		Positive Samples		Total Samples	
	N	%	N	%	N	%
2017	40	43.5	52	56.5	92	100
2018	39	47.0	44	53.0	83	100
2019	37	49.3	38	50.7	75	100
2020	39	49.4	40	50.6	79	100
2021	44	50.6	43	49.4	87	100
2022	35	44.9	43	55.1	78	100
Total	234		260		494	

^1^ The significance level for the distribution of negative and positive samples for *Salmonella* spp. in relation to the year (*p*-value = 0.92 > 0.05). Abbreviations: N, number of samples.

**Table 2:** The annual proportion of samples tested positive for *Salmonella* spp. in each municipality of Quindío.

Municipality	Year					
	2017 +/N (%)	2018 +/N (%)	2019 +/N (%)	2020 +/N (%)	2021 +/N (%)	2022 +/N (%)
Armenia	17/25 (68)	9/15 (60)	9/14 (64)	9/13 (69)	14/19 (73)	10/13 (76)
Buenavista	2/3 (66)	1/4 (25)	2/4 (50)	1/3 (33)	1/3 (33)	2/4 (50)
Calarcá	7/12 (58)	7/12 (58)	7/13 (53)	7/13 (53)	8/15 (53)	8/13 (61)
Circasia	3/5 (60)	3/5 (60)	3/5 (60)	3/5 (60)	1/3 (33)	2/3 (66)
Córdoba	0/1 (0)	0/1 (0)	0/1 (0)	0/1 (0)	0/1 (0)	0/1 (0)
Filandia	3/5 (60)	4/6 (66)	1/3 (33)	3/6 (50)	2/5 (40)	2/5 (40)
Génova	1/3 (33)	1/3 (33)	0/2(0)	0/2(0)	0/3(0)	0/2(0)
La Tebaida	3/7 (42)	2/6 (33)	1/4 (25)	2/5 (40)	2/6 (33)	2/6 (33)
Montenegro	4/10 (40)	5/10 (50)	5/11 (45)	5/11 (45)	3/10 (30)	5/11 (45)
Pijao	1/2 (50)	1/2 (50)	1/2 (50)	1/2 (50)	1/2 (50)	2/3 (66)
Quimbaya	7/13 (53)	5/10 (50)	4/8 (50)	5/11 (45)	5/11 (45)	5/10 (50)
Salento	4/6 (66)	6/9 (66)	5/8 (62)	4/7 (57)	6/9 (66)	5/7 (71)

Abbreviations: +, number of positive samples; N, total number of samples; %, proportion of positive samples.

**Table 3:** The proportion of ready-to-eat artisanal sausage samples positive and negative for *Salmonella* spp. in the different food outlets.

Ready-to-Eat Food Outlets ^1^	Negative Samples		Positive Samples		Total Samples	Prevalence
	N	%	N	%		(%)
Restaurants	95	40.6	41	15.8	136	30.1
Quick-service restaurants	66	28.2	67	25.8	133	50.4
Street vendors	73	31.2	152	58.4	225	67.6
Total	234	100	260	100	494	

^1^ The significance level for the distribution of negative and positive samples for *Salmonella* spp. in relation to food outlets (*p*-value = 0.00001 < 0.05). Abbreviations: N, number of samples.

**Table 4:** The proportion of ready-to-eat artisanal sausage samples positive and negative for *Salmonella* spp. according to types of cooking.

Type of Cooking ^1^	Negative Samples		Positive Samples		Total Samples	Prevalence
	N	%	N	%		(%)
Smoked barbecue	79	33.8	179	68.8	258	69.4
Grilled	56	23.9	47	18.1	103	45.6
Fried	99	42.3	34	13.1	133	25.5
Total	234	100	260	100	494	

^1^ The significance level for the distribution of negative and positive samples for *Salmonella* spp. in relation to food outlets (*p*-value = 0.00003 < 0.05). Abbreviations: N, number of samples.

## Data Availability

The datasets generated during and/or analyzed during the current study are available from the corresponding author on reasonable request.
